# Pai Syndrome: Median Cleft Lip, Corpus Callosum Lipoma, and Fibroepithelial Skin Tag

**Published:** 2014-03-15

**Authors:** Jacob Azurdia, Leah Burke, Donald Laub

**Affiliations:** University of Vermont College of Medicine, Burlington, Vt

**Keywords:** Pai syndrome, median clef t lip, corpus callosum dysgenesis, Fibroepithelial skin polyps, lipoma

## DESCRIPTION

**Figure 1:**
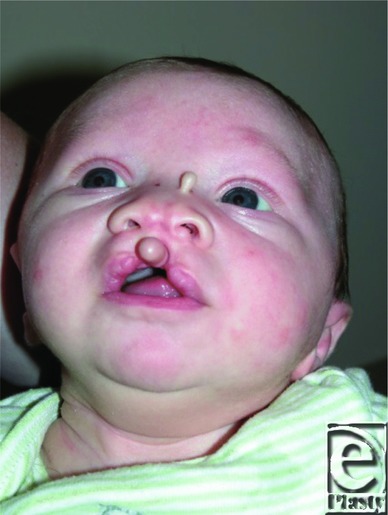
Presentation with median cleft lip, widened nasal tip, and fibroepithelial skin polyps.

**Figure 2:**
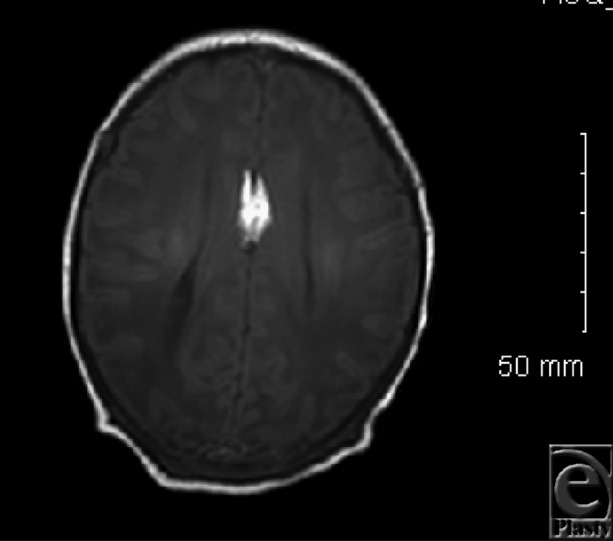
Magnetic resonance imaging demonstrating dysgenesis and lipoma of the corpus callosum.

The patient is a male infant with a midline cleft lip, with widened nasal tip, and fibroepithelial skin polyps of the nasal dorsum and philtral midline (Fig 1). He also has a lipoma of the interhemispheric fissure, and dysgenesis of the corpus callosum (Fig 2).

## QUESTIONS

1. **What are the manifestations of Pai Syndrome?**
2. **What are the embryogenic components of median cleft lip?**
3. **What are animal models of median cleft lip?**
4. **What is the surgical management of median cleft lip?**

## DISCUSSION

Median clefts are a rare craniofacial anomaly, effecting 0.2% of patients with orofacial clefting.^1^ Median cleft lip has been associated with various manifestations of midline dysplasia; one relatively common finding is cutaneous polyps attached in the nose, lip skin, or alveolus.^2^ Our patient had a skin polyp in the cleft midline, suggesting the appearance of the prolabium of a bilateral cleft lip on casual inspection (Fig 1). Masuno et al described a case of median cleft lip with cutaneous polyps and a chromosomal reciprocal translocation.^3^ Pai syndrome was first described in 1987 expressed as a midline orofacial cleft, cutaneous polyps, and lipoma of the corpus callosum.^4^ The corpus callosum may have varying degrees of dysgenesis.^5^ Developmental delay is only occasionally seen in this syndrome, and usually it is related to other associated condition, rather than the Pai syndrome per se.^4^^,^^5^

The genetic orchestration of the formation of the upper lip is still being elucidated. Genes and molecular pathways that are involved in the regulation of neural crest formation, migration, patterning, proliferation, and apoptosis appear to be important players. In the *Wnt* pathway, *Wnt3* and *Wnt9b* are particularly expressed in the underlying mesenchyme of the medial nasal prominences and lateral nasal prominences.^6^

The natural median groove in a mouse's upper lip is somewhat similar to the median clefts seen in Pai syndrome. The order of fusion of the maxillary and nasal processes differs in mice compared to humans, but studies in mice employing antagonists of the Hedgehog pathway to induce the formation of cleft lip (both lateral and median) implicate the Sonic Hedgehog pathway as another important player in the development of the median cleft lip. A study using the frog as an animal model demonstrated that inhibiting retinoic acid synthesis enzyme (RALDH) induced median clefts in developing embryos. Furthermore, homeobox genes *Msx2* and *Lhx8* were shown to be regulated by *RARγ*. When both *RARγ* and homeobox enzymes *Msx2/Lhx8* were inhibited, median cleft lip was induced in 100% of test animals.^7^^,^^8^

**Figure 3:**
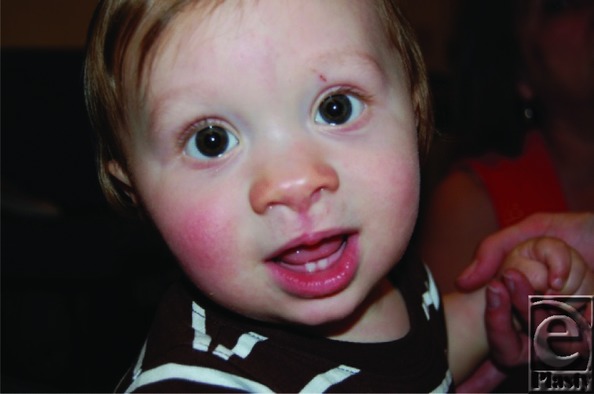
Good surgical result with straight-line closure.

Our patient had a surgical repair of his facial defect with a midline inverted “V” excision extending into the nasal tip, excision of the cutaneous polyps, and a straight-line closure. He had an acceptable surgical outcome (Fig 3) and has shown essentially normal neurologic development.
